# Supplementary material for: Soil moisture-evaporation coupling shifts into new gears under increasing CO2
Source: Nat Commun. 2023 Mar 1;14:1162. doi: 10.1038/s41467-023-36794-5 (PMC9977744; doi:10.1038/s41467-023-36794-5)
Supplement: Supplementary file 1 — Supplementary Information [file 41467_2023_36794_MOESM1_ESM.pdf]

Supplementary Information for

Soil moisture-evaporation coupling shifts into new gears under increasing CO_2

Hsin Hsu[1] (hhsu@gmu.edu) and Paul A. Dirmeyer[1,2]

[1]George Mason University, Fairfax, VA, USA

[2]Center for Ocean-Land-Atmosphere Studies, George Mason University, Fairfax, VA, USA

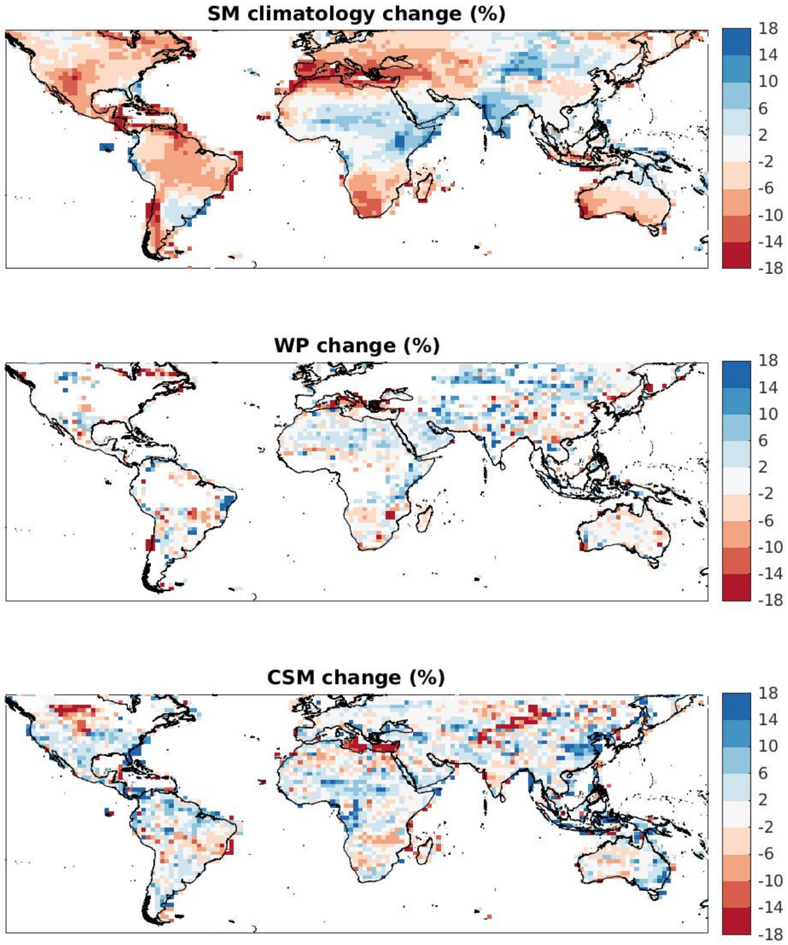

Figure S1. The ensemble mean of the percentage changes for (a) climatological soil moisture, (b) wilting point, and (c) critical soil moisture.

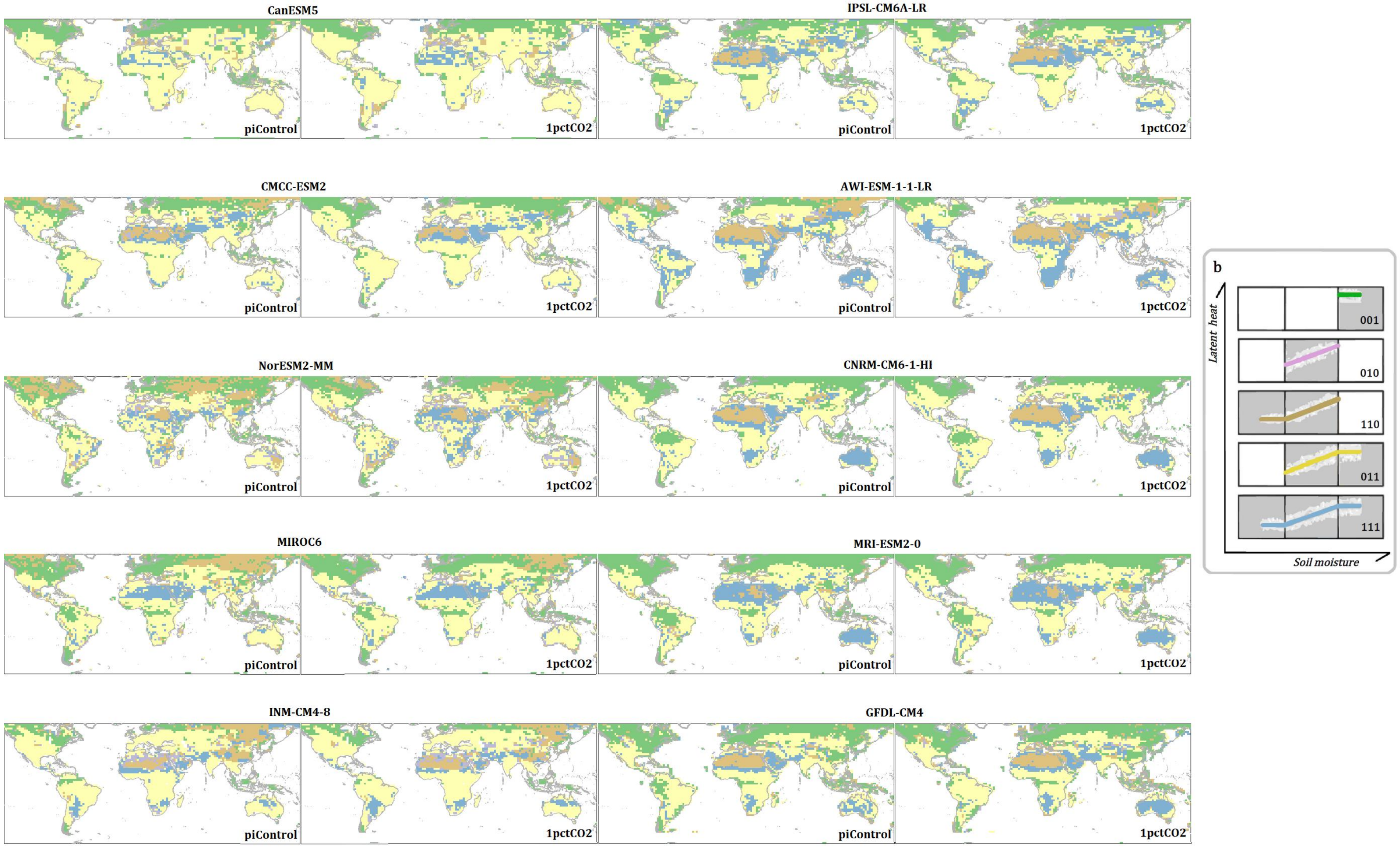

Figure S2. Global distribution of soil moisture regimes and their shifts under global warming for individual climate models in pre-industrial climate (piControl) and warming climate (1pctCO2).

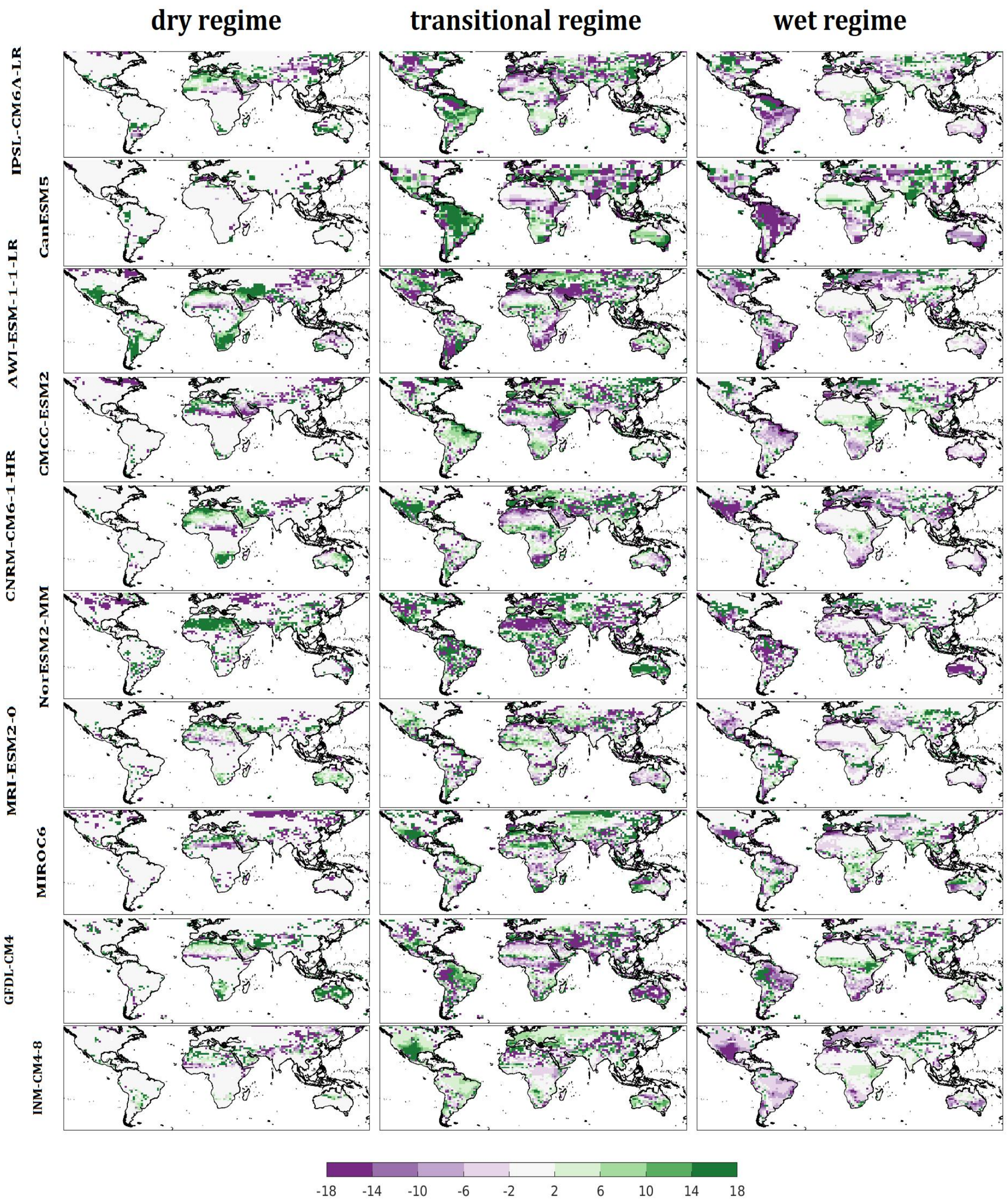

Figure S3. Change from piControl to 1pctCO2 in the fraction of days each model (rows) spends with SM in the indicated regime (columns), expressed as percentages (fraction × 100).

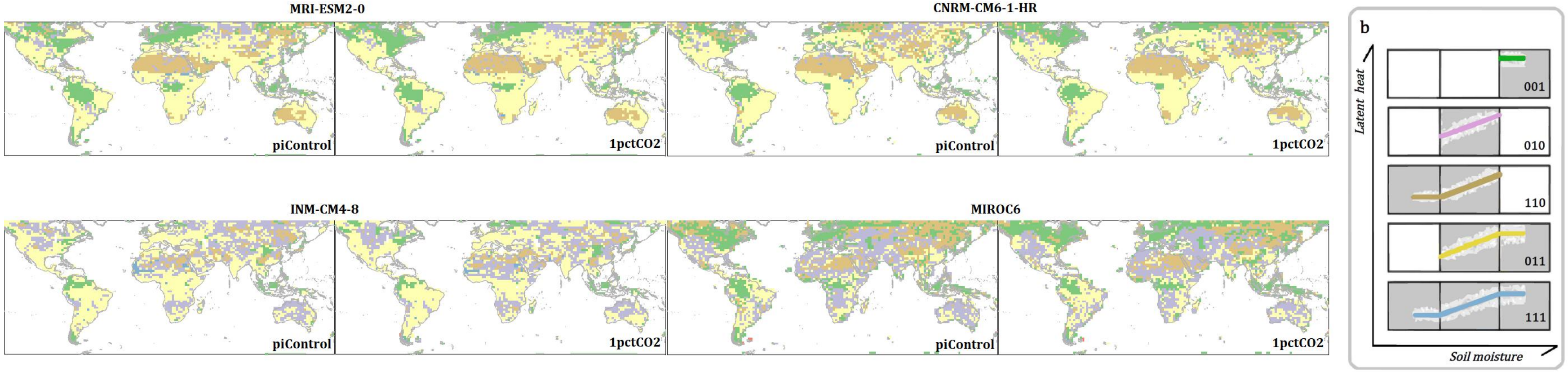

Figure S4. Global distribution of soil moisture regimes and their shifts under global warming but analysis is applied using total soil moisture (mrso) to replace surface soil moisture (mrsos) for the same set of experiments (Only data from MRI-ESM-0, CNRM-CM6-1-HR, INM-CM4-8, and MIROC6 were available at the time analysis was conducted).

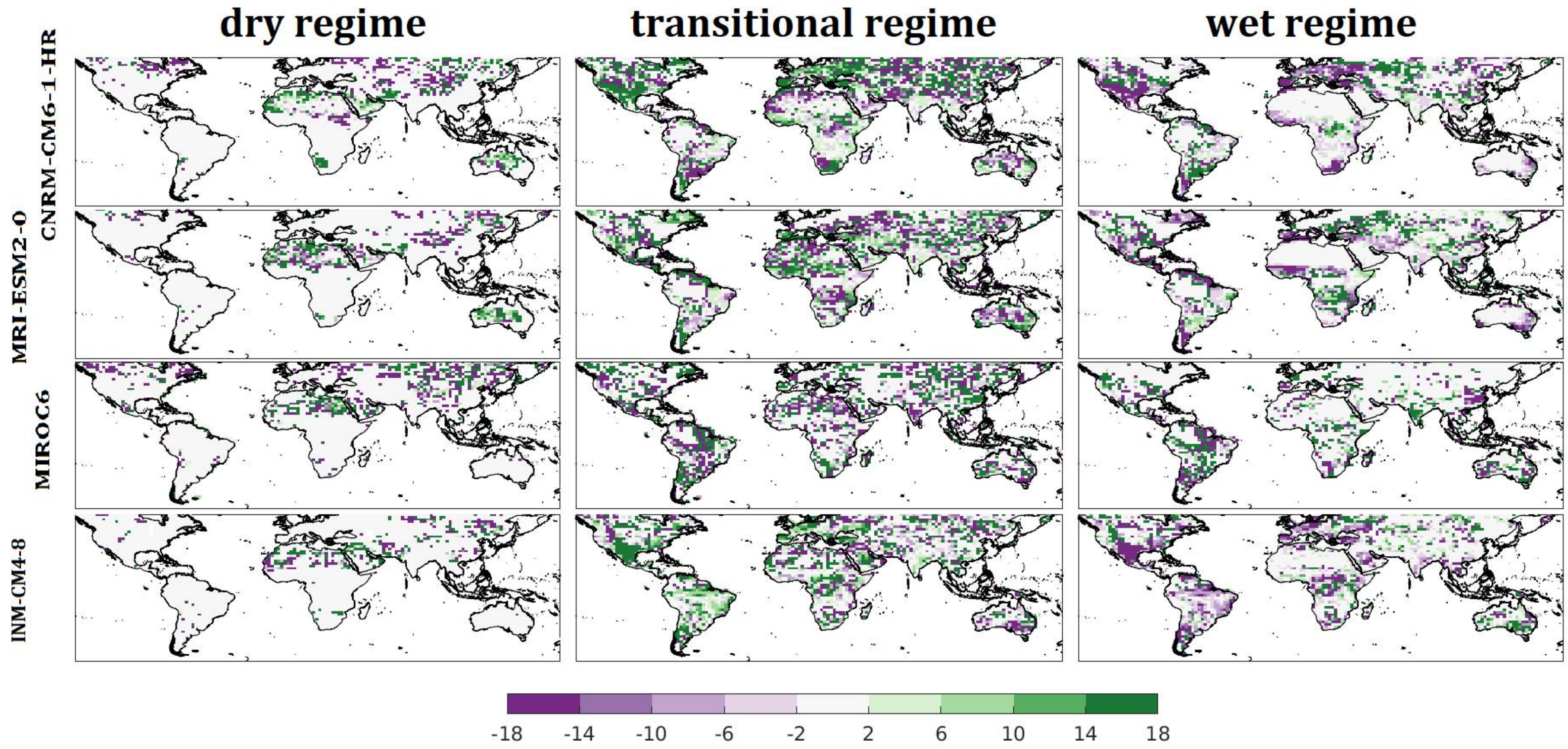

Figure S5. Change from piControl to 1pctCO2 in the fraction of days each model (rows) spends with SM in the indicated regime (columns), expressed as percentages (fraction × 100) but for total soil moisture (mrso) instead of surface soil moisture (mrsos).

Table S1. An example of how the mode among models is obtained at a grid cell (18°N, 14°E) from soil moisture regimes in pre-industrial climate and warming climate, determined by the best-fit segmented regression candidates identified in the breakpoint analysis (2nd and 3rd columns). The last column shows the main migration tendency of soil moisture between soil moisture regimes for each climate model (depicted in Fig.S3). If the changes within none of the soil moisture regimes, as calculated from the data shown in Fig.S3, is statistically significant, the model is tagged as NA.

Location: 18°N,14°E	Candidate of pre-industrial climate	Candidate of warming climate	Migration (Shift from/ Shift to)
MIROC6	C110	C111	Dry/Transitional
AWI-ESM-1-1-LR	C011	C011	NA
CMCC-ESM2	C111	C111	Dry/Transitional
CanESM5	C111	C111	Dry/Transitional
CNRM-CM6-1-HR	C110	C110	NA
NorESM2-MM	C111	C111	Transitional/Dry
IPSL-CM6A-LR	C110	C111	Dry/Transitional
MRI-ESM2-0	C111	C111	Dry/Transitional
GFDL-CM4	C110	C110	NA
INM-CM4-8	C110	C110	NA
Mode	C110	C111	Dry/Transitional

Table S2: CMIP model data citations, resolutions (RES; unit: degree), and the period of each simulation used for analysis

CMIP Label	RES (lat x lon)	Model year piControl	1pctCO2	Full Citation
AWI-ESM-1-1-LR	1.875x1.875	1880-1929	1950-1999	Semmler, T., & Co-authors (2018). AWI AWI-CM1.1MR model output prepared for CMIP6 CMIP. Earth System Grid Federation. doi: https://doi.org/10.22033/ESGF/CMIP6.359.
CNRM-CM6-1-HR	0.25x0.25	1880-1929	1950-1999	Voldoire, A. (2018). CNRM-CERFACS CNRM-CM6-1 model output prepared for CMIP6 CMIP. Earth System Grid Federation. doi: https://doi.org/10.22033/ESGF/CMIP6.1375.
IPSL-CM6A-LR	1.25x2.5	1880-1929	1950-1999	Boucher, O.; Denvil, S.; Caubel, A.; Foujols, M. A. (2020). IPSL IPSL-CM6A-LR-INCA model output prepared for CMIP6 AerChemMIP. Earth System Grid Federation. doi: https://doi.org/10.22033/ESGF/CMIP6.13581.
MIROC6	1.4x1.4	3300-3349	3300-3349	Takemura, T. (2019). MIROC MIROC6 model output prepared for CMIP6 AerChemMIP. Earth System Grid Federation. doi: https://doi.org/10.22033/ESGF/CMIP6.9121.
CMCC-ESM2	0.9375x1.25	1880-1929	1950-1999	Lovato, T., & Butenschön, M. (2021). CMCC CMCC-ESM2 model output prepared for CMIP6 OMIP (Version 20210127). Earth System Grid Federation. https://doi.org/10.22033/ESGF/CMIP6.13167
CanESM5	2.8125x2.8125	5250-5249	1950-1999	Swart, N. C., & Co-authors (2019). CCCma CanESM5 model output prepared for CMIP6 CMIP. (Version 20190502).Earth System Grid Federation. https://doi.org/10.22033/ESGF/CMIP6.1303
NorESM2-MM	0.9375x1.25	1230-1279	0100-0149	Bethke, I. & Co-authors (2019). NCC NorCPM1 model output prepared for CMIP6 CMIP. Earth System Grid Federation. doi: https://doi.org/10.22033/ESGF/CMIP6.10843.
MRI-ESM2-0	1.125x1.125	1880-1929	1950-1999	Yukimoto, S. & Co-authors (2019). MRI MRI-ESM2.0 model output prepared for CMIP6 CMIP. Version.Earth System Grid Federation. https://doi.org/10.22033/ESGF/CMIP6.621
GFDL-CM4	1x0.8	0275-324	0100-0150	Guo, Huan;& Co-authors (2018). NOAA-GFDL GFDL-CM4 model output..Earth System Grid Federation. https://doi.org/10.22033/ESGF/CMIP6.1402
INM-CM4-8	1.5x2	1880-1929	1950-1999	Volodin, Evgeny & Co-authors (2018)(2019). INM INM-CM4-8 model output prepared for CMIP6 CMIP piControl. Earth System Grid Federation. https://doi.org/10.22033/ESGF/CMIP6.5080
